# Understanding older people's voice interactions with smart voice assistants: a new modified rule-based natural language processing model with human input

**DOI:** 10.3389/fdgth.2024.1329910

**Published:** 2024-05-14

**Authors:** Zhengxu Yan, Victoria Dube, Judith Heselton, Kate Johnson, Changmin Yan, Valerie Jones, Julie Blaskewicz Boron, Marcia Shade

**Affiliations:** ^1^ College of Computing, Data Science and Society, University of California-Berkeley, Berkeley, CA, United States; ^2^ Department of Gerontology, University of Nebraska-Omaha, Omaha, NE, United States; ^3^ College of Journalism and Mass Communications, University of Nebraska-Lincoln, Lincoln, NE, United States; ^4^ College of Nursing, University of Nebraska Medical Center, Omaha, NE, United States

**Keywords:** natural language processing, aging, gerontology, artificial intelligence, smart voice assistants (SVA), algorithms, coding, content analysis

## Abstract

The COVID-19 pandemic has expedited the integration of Smart Voice Assistants (SVA) among older people. The qualitative data derived from user commands on SVA is pivotal for elucidating the engagement patterns of older individuals with such systems. However, the sheer volume of user-generated voice interaction data presents a formidable challenge for manual coding. Compounding this issue, age-related cognitive decline and alterations in speech patterns further complicate the interpretation of older users’ SVA voice interactions. Conventional dictionary-based textual analysis tools, which count word frequencies, are inadequate in capturing the evolving and communicative essence of these interactions that unfold over a series of dialogues and modify with time. To address these challenges, our study introduces a novel, modified rule-based Natural Language Processing (MR-NLP) model augmented with human input. This reproducible approach capitalizes on human-derived insights to establish a lexicon of critical keywords and to formulate rules for the iterative refinement of the NLP model. English speakers, aged 50 or older and residing alone, were enlisted to engage with Amazon Alexa™ via predefined daily routines for a minimum of 30 min daily spanning three months (*N* = 35, mean age = 77). We amassed time-stamped, textual data comprising participants’ user commands and responses from Alexa™. Initially, a subset constituting 20% of the data (1,020 instances) underwent manual coding by human coder, predicated on keywords and commands. Separately, a rule-based Natural Language Processing (NLP) methodology was employed to code the identical subset. Discrepancies arising between human coder and the NLP model programmer were deliberated upon and reconciled to refine the rule-based NLP coding framework for the entire dataset. The modified rule-based NLP approach demonstrated notable enhancements in efficiency and scalability and reduced susceptibility to inadvertent errors in comparison to manual coding. Furthermore, human input was instrumental in augmenting the NLP model, yielding insights germane to the aging adult demographic, such as recurring speech patterns or ambiguities. By disseminating this innovative software solution to the scientific community, we endeavor to advance research and innovation in NLP model formulation, subsequently contributing to the understanding of older people's interactions with SVA and other AI-powered systems.

## Introduction

Older individuals have increasingly adopted AI-powered smart voice assistants (SVA) in recent years, preferring speech-based hands-free and eyes-free interactions over typing or clicking modalities (1–3). SVA employs natural language processing (NLP) AI techniques to interact with user commands. Aiming to understand older people's SVA usage and its effects, researchers often use a dictionary-based text analysis of keywords in qualitative user commands recorded on SVA to gain insights into aspects like anthropomorphizing SVA or ethical considerations related to AI technology (4–6). However, such a dictionary-based approach oversimplifies older people's interactions with SVA to a one-way initiative by the user and fails to capture the communicative nature of such interactions—the transmission and coordination of complex information with others (7).

Interactions with SVA often evolve over multiple conversations. Unlike simple one-off commands or queries, users gradually develop a rapport with their assistants, fine-tuning their preferences and refining their requests (5). Over time, they might engage in follow-up questions, seek clarifications, or adjust instructions based on prior interactions. This iterative dialogue process mirrors the natural progression of human conversations, where understanding deepens and adjusts over repeated exchanges. Existing dictionary-based text analysis tools, such as Linguistic Inquiry and Word Count (LIWC), can only account for word frequencies of a conversation, not episodes of distinct conversations (8). The widely used LIWC cannot capture the dynamic nature of these SVA interactions and fail to showcase the potential of voice assistants to adapt, learn, and provide more personalized assistance as they engage with users over time.

In addition, as people age, they often experience a decline in cognitive abilities and undergo changes in their speech patterns (9). Consequently, older individuals might not always interact with AI-powered SVA through just one clear command. Instead, their interactions might involve multiple repetitions of commands, corrections, and confirmations. This pattern underscores the unique challenges and considerations when analyzing textual data of older people's interactions with SVA.

In this research, we introduce an innovative hybrid natural language processing (NLP) framework, integrating a modified rule-based NLP (MR-NLP) model with human input. This platform independent, replicable approach synergizes manual and automated coding techniques to analyze the voice interaction data of older individuals with Smart Voice Assistants (SVA). The methodology incorporates human-derived insights to construct a lexicon of pivotal keywords and guide iterative modifications of the NLP model, ensuring it effectively captures the nuanced dialogue dynamics in older adults’ engagements with SVA. Our objective is to employ a large-scale data analysis strategy to elucidate the nature of older adults’ communication with AI-enhanced SVA. Through this innovative MR-NLP software solution, we aim to significantly advance NLP model development, focusing on enhancing data handling capacity, processing efficiency, accuracy, scalability, and the ability to interpret and account for contextual meanings within the textual data of old people's voice interactions with SVA. In addition to replicating our proposed MR-NLP framework, researchers can access an online web app version (a.k.a., Automated Textual Data Analysis) with permission via https://excel-helper-deploy.vercel.app.

### Adoption of SVA by older people

The adoption and usage of Smart Voice Assistants (SVA), such as Amazon Alexa™, Google Assistant™, or Apple Siri™, have experienced widespread popularity in recent years. These intelligent voice assistants with natural language processing technologies offer a user-friendly interface, requiring minimal technological expertise, thus making them accessible to and usable for older adults with varying levels of digital literacy (10, 11, 12). As older individuals seek ways to improve communication with family members, enjoy entertainment, and engage socially, many are turning to AI-powered SVA devices (13, 14), gradually replacing traditional information and communication technologies (ICT) like online platforms, email, smartphones, and iPads.

According to the Urban Institute, the number of Americans aged 65 and older will double in the next 40 years, hitting 80 million by 2040. Meanwhile, the group of adults aged 85 and older, who most frequently require assistance with basic personal care, will see their numbers increase nearly fourfold from 2000 to 2040. Many older Americans are starting to use smart assistant technologies to aid their daily routines and personal care (15).

According to a 2021 survey conducted by the American Association of Retired Persons (AARP) among adults aged 50 and above, approximately 82% of them use technology as a means of staying connected with family and friends. Furthermore, around 40% of this age group utilize smart devices, including smartphones and voice assistants, to communicate with medical practitioners for various healthcare purposes, such as telehealth consultations, prescription orders, receiving personalized medical advice, and scheduling appointments (16). Research suggests using SVAs can even help older adults improve their health, reducing perceptions of loneliness and pain, and increasing social support and social connectedness (10, 11, 17).

The adoption of technology in healthcare management among older adults is often influenced by factors like cognitive ability, perceived ease of use, technological self-efficacy, and social influence (1–3). The hands-free and eyes-free interaction with SVA provides older individuals with immediate and intuitive benefits, especially when considering physical challenges (1, 3). The ease of use can be critical, as more interaction with NLP-powered SVAs may lead to greater health benefits (18). However, this also brings about new difficulties in understanding their verbal interactions with the SVA.

### Age-related changes in cognitive capacities and speech patterns

The cognitive decline experienced by older individuals often correlates with a decrease in the complexity and diversity of their linguistic output (19). Changes in speech patterns, including verbal repetitions, pauses, and slowed speech, have been suggested as potential early indicators of cognitive impairment (20). Furthermore, older people with cognitive impairment may encounter difficulties in understanding speech (21). Consequently, older individuals’ interactions with SVA may be influenced by declining cognitive capacities, leading to speech repetitions, interruptions, or irregularities. These factors can pose challenges when processing qualitative human-SVA speech data. In interventions spanning several months, generating substantial quantities of human-AI voice interaction data, there is a higher risk of misinterpretation or misplacement of codes. Therefore, an alternative method for coding qualitative human-AI voice interaction data is necessary to ensure accurate and reliable analysis.

### Coding of human-SVA speech interaction data

Human-SVA speech interaction data is often subject to manual coding using thematic analysis (22) to identify patterns or themes (3, 5). However, manual coding of speech data can present potential challenges, including subjectivity, time and labor intensiveness, and human fatigue (23, 24), which can be exacerbated by the large volumes of textual data generated through human-SVA interactions. Dictionary-based text analysis tools, such as the Linguistic Inquiry and Word Count (LIWC), have been used in computational linguistics (25) and psychology (26) for quantifying large volumes of textual information. LIWC maps words in a text to categories predefined in a dictionary. Each category represents a psychological construct, emotion, or thematic element. While LIWC automates categorizing and counting pre-defined keywords, it cannot account for variances in textual data that are not listed in the dictionary and isn't meant to capture contextual nuances and episodical themes in conversational data.

Decoding human-SVA interactions often involves complex linguistic tasks such as context understanding and disambiguation that go beyond simple word counts. Natural Language Processing (NLP) techniques are appropriate tools for such tasks. NLP with either human-developed rules or machine learning has been developed to automate the manual coding process by extracting textual phrases that indicate thematic concepts of interest (27, 28). While rule-based NLP relies on expert-crafted linguistic rules to extract structured information (29), machine learning based NLP uses statistical models to automate text classification (30). Both NLP approaches yield rapid results of textual data coding, significantly saving on labor and costs (31, 32).

While both NLP methods can automate manual coding, rule-based NLP is better suited for processing human-SVA speech interaction data, particularly due to considerations of sample size. Past studies on older people's use of SVA often involve small sample sizes (e.g., less than 100) and don't meet the threshold of an adequate effect size for machine learning-based NLP, i.e., 0.5 or higher according to Cohen's scale (33). In contrast, rule-based NLP can effectively process qualitative textual data from small sample sizes, requiring only an NLP expert to develop rules (28). Therefore, we propose a new protocol for developing a rule-based NLP framework to capture the nuances of older people's conversations with SVA. The methodology we propose leverages human contributions to formulate a lexicon of pivotal keywords and expert-based rules to guide iterative refinements in the NLP model. To demonstrate the superiority of our MR-NLP model over the manual coding of older people's conversational data with SVA in previous studies (3, 5), the following hypotheses are proposed to test data processing efficiency and accuracy.

*H1: Modified Rule-based NLP (MR-NLP) coding of human-SVA speech interaction data will demonstrate higher efficiency compared to manual coding of the same data*.

*H2: Modified Rule-based NLP (MR-NLP) coding of human-SVA speech interaction data will exhibit fewer unintentional errors than manual coding of the same data*.

*H3: Modified Rule-based NLP (MR-NLP) coding of human-SVA speech interaction data will result in more accurate analytical interpretations than manual coding of the same data*.

## Method

### Sampling and recruitment

To be considered for inclusion in this study, participants were required to meet several criteria aimed at ensuring their ability to engage with and benefit from the study among older adults. Specifically, eligibility criteria stipulated that participants must be fluent in English, reside alone in an independent living facility within the region, be aged 50 years or older, and have a history of engaging with smart speakers—specifically, Amazon Echo or Google Nest devices—no more than three times per week over the last 30 days. Additionally, participants were required to demonstrate a willingness to interact with an Amazon Alexa™ SVA for a minimum of 30 min daily over a 12-week period and to successfully complete a capacity to consent assessment administered by the research team.

The study recruited a cohort of 34 participants (38% male, 62% female; mean age = 77 years, SD = 11.52, range: 50–98 years; 94% White, 3% African American, 3% Asian American). This sample was selected to explore older adults’ engagement in SVA-based daily routines, such as greetings, accessing weather forecasts, participating in digital games, and making calls to maintain social connections.

The Montreal Cognitive Assessment (MoCA) (34) was administered by MoCA-certified members of the research team prior to the study's commencement to gauge participants’ cognitive function, yielding an average score of 23.47 (SD = 3.65, score range: 14–30). Using a threshold score of 26 for mild cognitive impairment, it was observed that 26% of the study participants attained scores equal to or surpassing this benchmark on the MoCA. This assessment was critical for understanding the cognitive baseline of participants, acknowledging that the sample might include individuals with varying levels of cognitive impairment.

The utilization of the MoCA within our research was strictly limited to descriptive objectives aimed at characterizing the participant sample. The MoCA has a maximum score of 30. Individuals scoring 26 and above are considered to have normal cognitive functioning (for individuals aged 55 + years). Those scoring between 18 and 25 points may be considered to show signs of mild cognitive impairment (MCI), whereas those scoring below 17 may show signs of more significant cognitive impairment. It is imperative to underscore, however, that the MoCA score, in isolation, does not suffice for a clinical diagnosis of MCI or dementia; it represents merely one of several diagnostic indicators. In the context of our investigation, the MoCA was employed solely as an ancillary measure for sample description, without influencing participant inclusion or exclusion based on their MoCA performance. Additionally, it is noteworthy that all participants underwent a capacity to consent assessment and provided informed consent prior to the MoCA administration. Although all research personnel involved were trained in the administration of the MoCA, it is important to clarify that they do not possess the clinical qualifications necessary for diagnosing MCI or dementia.

To ensure the study was tailored to accommodate the diverse cognitive capabilities within the sample, steps were taken to make engagement with the SVA activities as inclusive and accessible as possible. Drawing upon user reviews and ratings available on Alexa, a panel comprising experts in geriatrics research, along with scholars and practitioners in media and technology, selected ten daily routines (see Supplementary Material Appendix 1 for detail descriptions). These routines were chosen based on their widespread popularity, inherent simplicity, and the ease with which they can be activated via simple voice command. These routines encompassed a broad range of activities designed to stimulate engagement, offer entertainment, and facilitate connection. The selection included various forms of entertainment such as music, jokes, games (including Akinator), riddles, and meditation practices like Five Minute Morning. It also comprised informational activities, for instance, weather updates through Big Sky, alongside features aimed at social interaction, such as greetings and the facilitation of phone calls. This approach was adopted to ensure that regardless of individual cognitive differences, every participant could find value and ease in engaging with the SVA activities.

Recruitment was conducted through direct outreach to independent living facilities across the region. Facilities were selected based on their residents’ demographic alignment with the study's target population, and within each facility, residents meeting the eligibility criteria were invited to participate. The recruitment and study procedures received approval from the University of Nebraska-Lincoln Institutional Review Board (IRB #20220321416FB), and all participants provided informed consent.

### Manual coding

Participants’ user commands and Amazon Alexa™ responses were recorded and extracted from their individual Amazon SVA accounts. The original raw data consist of time-stamped, text-based commands and responses, organized chronologically in Excel files. Given that the textual data of participants’ user commands to Alexa are embedded within each participant's dataset, a methodologically sound approach was adopted to select a representative subset for manual coding. Specifically, a random sampling technique was applied to select 20% of the participant pool, equating to 7 participants, as a manageable subset for in-depth manual coding analysis. This selection strategy was planned to achieve a balance between the intensive labor required for manual coding, entailing 20 h dedicated to the development of a codebook and an additional 15 h for coding, and the need for a robust dataset sufficient for analytical scrutiny, including the examination of daily routines and other interactions with Alexa.

For the initial analysis, the 20% subset (*n* = 7) of the data (1,020 cases) was manually coded by human coder. In examining voice-activated device interactions, a case can be a scenario ranging from a singular command, such as “Alexa, play country music for five minutes,” to complex command sequences for interactive experiences like the Akinator game on Amazon Alexa. Akinator, a voice-controlled adaptation of the popular web game for Alexa-enabled devices, utilizes advanced voice recognition to engage users in interactive storytelling. In this game, the genie, Akinator, guesses characters thought of by users through a series of yes-or-no questions. Interactions typically involve initiating the game with a command and responding to the genie's questions with a sequence of answers like “Yes,” “No,” “I don't know,” and “Yes.” A set of coding standards were developed to accounted for successful completions of daily routines and other meaningful interactions (see Supplementary Material Appendix 2), using keywords and commands to group them into 12 distinct daily routines and other interactions such as entertainment (e.g., music, joke, game/Akinator, riddle, meditation/Five Minute Morning), information (e.g., weather/Big Sky), greetings, calls, settings, and additional interactions not part of the pre-programmed daily routines.

To evaluate the representativeness of this subset, independent sample t-tests were conducted to compare demographic and cognitive characteristics (namely, gender: t(33)=−0.17, *p* = 0.88; age: t(33) = 0.46, *p* = 0.65; race: t(33)=−0.69, *p* = 0.49; MoCA scores: t(33) = 0, *p* = 1.00) and the 12 categories of daily routines and other interactions (between the selected 7-participant subset and the entire cohort of 35 participants. The statistical analysis, conducted at an alpha level of.05, revealed no significant differences among any participant characteristics or SVA interactions (Good Morning: t(33) = 1.12, *p* = 0.27; Big Sky: t(33) = 0.97, *p* = 0.34; Riddle: t(33) = 1.41, *p* = 0.17; Five Minute Morning Meditation: t(33) = 1.27, *p* = 0.21; Music: t(33) = 0.31, *p* = 0.76; Good Afternoon & Good Evening: t(33) = 0.76, *p* = 0.45; Weather: t(33) = 0.96, *p* = 0.34; Joke: t(33) = 0.35, *p* = 0.73; Akinator: t(33) = 0.12, *p* = 0.90; Calls: t(33) = 0.11, *p* = 0.91; Good Night: t(33) = 1.19, *p* = 0.24; Setting Volume & Speed: t(33) = 0.34, *p* = 0.73), thereby substantiating the subset's representativeness of the broader dataset.

### Rule-based NLP coding

In line with the procedure outlined in the rule development approach of NLP (22), an expert NLP programmer reviewed the coding standards developed by the human coder and gained a comprehensive understanding of how the keywords and commands were coded. Subsequently, knowledge-based rules were applied to conduct NLP coding of the same raw data.

### Text normalization: chronological data sorting

**Figure 1:**
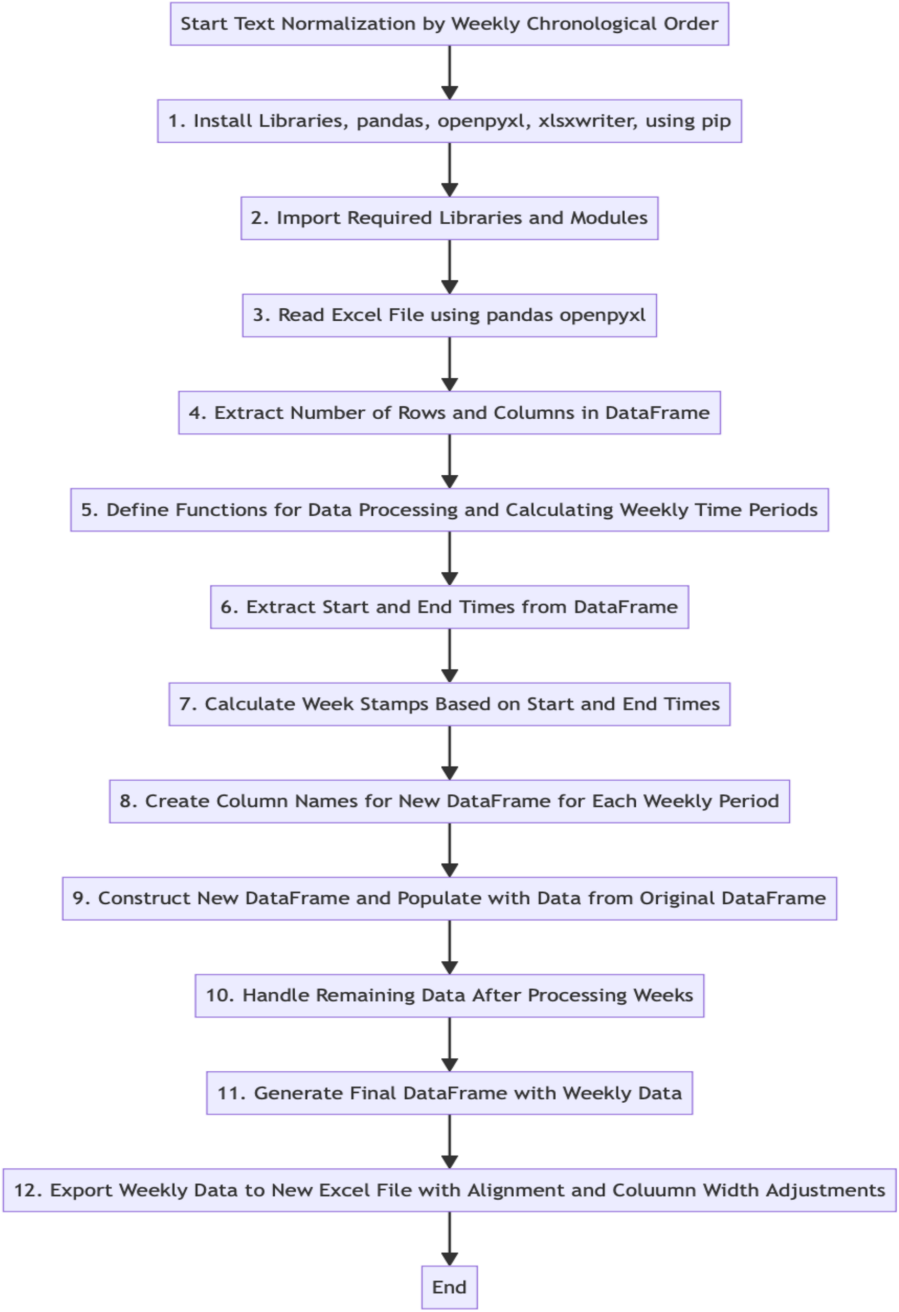
Text normalization by sorting the data by weeks.

To organize the raw data into a consistent chronological order, i.e., weekly segments, several libraries, including pandas, openpyxl, xlsxwriter, and the Python programming language, were utilized to read and process the same 20% subset (n = 7) of the data. The resulting weekly data was then stored in a new DataFrame to facilitate easy visualization and interpretation. The procedure for the text normalization by sorting the data by weeks is outlined in Figure 1, generated in *Mermaid v10.5.0 Live Editor*.

After the analysis of the downloaded data using Python, a new Excel file was generated, containing the weekly data. The weekly data was organized into columns representing each weekly period, while rows corresponded to different data points or observations for each participant. The program automatically divided the data into weekly segments within seconds, eliminating the need for manual intervention.

To validate the accuracy of the data sorting by weeks performed by the program, a comparison was made with the manual coding results based on a randomly selected participant's data. During this comparison, a discrepancy was identified in one instance—specifically, a single data cell of week 4 for this participant. According to the NLP program's categorization, it should have been classified under week 3, as indicated by the calendar date. After discussing with the human coder, it was determined that this discrepancy resulted from an oversight made by the human coder.

### Data sense-making using rule-based NLP or MR-NLP

Utilizing the same 12 categories of daily routines and other interactions as used in manual coding, the 20% subset dataset was processed using the Python programming language, employing an XML export feature and leveraging various libraries such as TextBlob and pandas. The TextBlob library proved invaluable for its capacity to execute robust text processing tasks, including word extraction and the identification of similarities and differences between user commands.

The goal was to utilize the rule-based NLP technique to extract meaningful insights from a dataset consisting of user commands and responses. To achieve this, a set of predefined keywords was established, and various functions were developed to identify these keywords within user inputs. These functions utilized text processing techniques, such as tokenization and word comparison, to determine the presence of keywords and categorize the commands accordingly. To account for the iterative dialogue process of user-SVA interactions, each successful completion of a routine or activity is defined by either a single command input execution or repeated command inputs in a linear continuous time frame. The results of the analysis were cross-tabulated, displaying the frequency of each keyword across different weeks. Additionally, an XML export feature enhanced data compatibility and integration.

**Figure 2:**
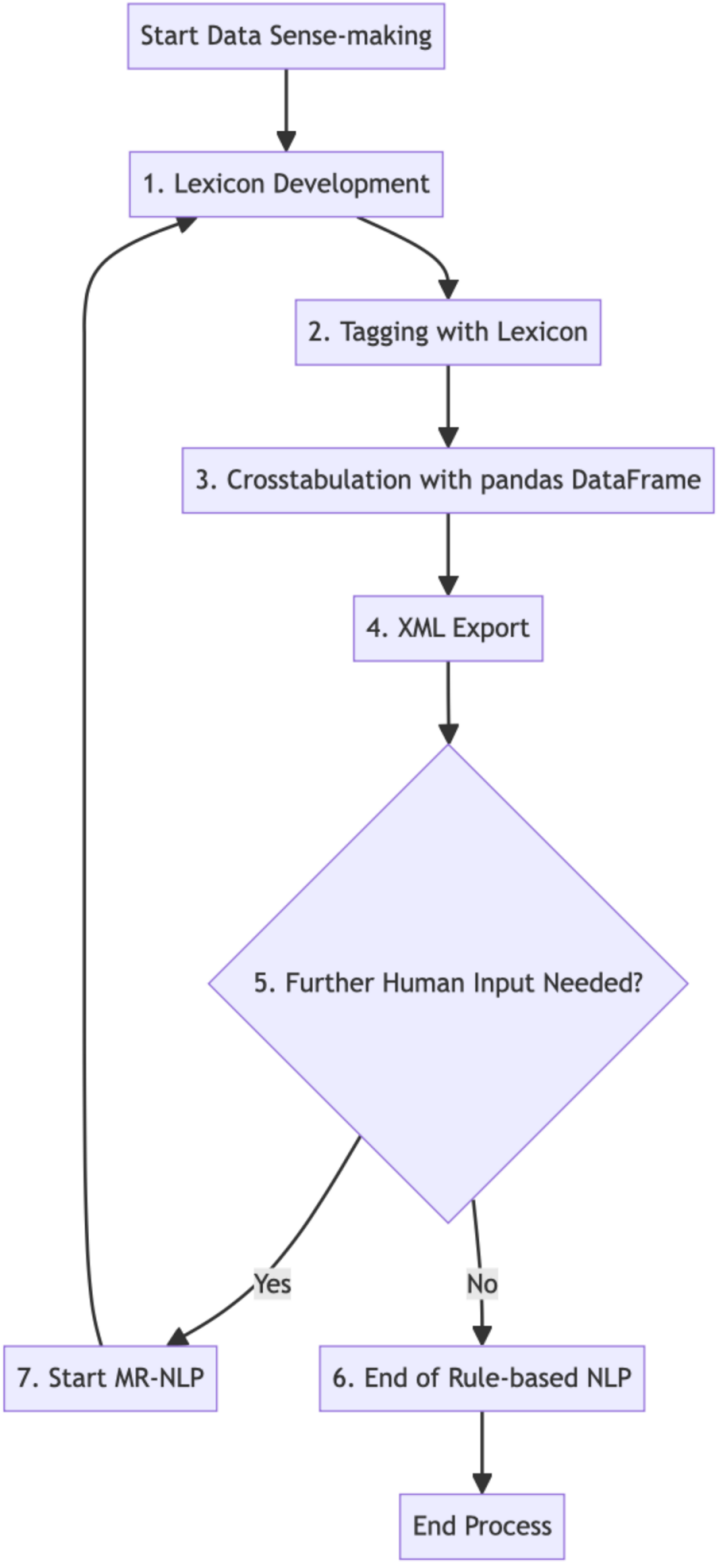
Data sense-making by rule-based NLP or modified rule-based NLP (MR-NLP).

Details of the rule-based NLP coding by categories of routines and other interactions are described below and illustrated in Figure 2 generated in *Mermaid v10.5.0 Live Editor*.

To start tagging using lexicon, a comprehensive set of predefined keywords was established, representing distinct types of commands. These keywords were organized systematically within an enumerated class referred to as “Keyword,” each assigned a corresponding numerical value. This classification system facilitated the straightforward identification and categorization of keywords.

Two crucial functions were developed to identify relevant keywords within user commands: “find_same_words” and “find_different_words.” The “find_same_words” function compared pairs of input strings and extracted any words that were identical in both, while the “find_different_words” function identified words present in one string but absent in the other. These functions enabled the identification of repeated commands, ensuring their accurate classification.

Furthermore, additional functions were implemented to specifically detect certain types of commands, such as Music, Weather, Akinator, Calls, and Setting_vol_speed. By utilizing a combination of keyword detection and contextual analysis, these functions determined the relevance and validity of each command, enabling more a focused analysis.

A pandas DataFrame named “word_df” was constructed to complete crosstabulation after the identification and categorization of keywords were completed. This DataFrame was structured with columns representing each keyword and rows corresponding to different weeks of user interactions. The frequency of each keyword within each week was recorded, allowing for the tracking of usage trends over time.

Additionally, an XML export feature was incorporated into the methodology. The “word_df” DataFrame was transformed into an XML format using the built-in XML exporting capabilities of the pandas library. This export process preserved the hierarchical structure of the DataFrame, facilitating further data analysis and integration with other tools or systems.

The methodology also involved data aggregation and the calculation of total keyword frequencies. By expanding the “word_df” DataFrame to include a row representing the total frequency of each keyword across all weeks, a comprehensive overview of the most used commands was obtained. This information served as a basis for in-depth analysis of user preferences and behaviors.

Human coder and NLP programmer discussed and resolved discrepancies. Based on knowledge learned from the discussion, the rule-based NLP technique was modified to code the entire data set among all 35 participants accordingly. In other words, 20% of the subset data set were coded by the original rule-based NLP model while the MR-NLP model incorporated insights learned from human coder and was applied to re-code the entire data.

Also, informed by discussions between the human coder and the NLP expert, the coding book (Supplementary Material Appendix 2) was revised to correct a primary source of human error coming from coding interactions with Akinator. The coding manual was updated to include the following guideline: “Repeated keywords or phrases within a single, continuous interaction should be considered as a single interaction.” Previously, human coders might overcount interactions with Akinator by tallying each occurrence of the keyword “Akinator.” For instance, a sequence including a command and responses to the genie's queries, such as “Alexa, play Akinator,” followed by “Yes,” “No,” “I don't know, Akinator,” and “Yes,” was mistakenly coded as two separate interactions. However, each set of interactions should be recognized as a continuous exchange marked by a series of affirmations or negations, accurately captured by the NLP model through considering the uninterrupted sequence of user engagement with the device.

In summary, the MR-NLP coding of categories of routines and other interactions proved effective in identifying, categorizing, and analyzing keywords and commands within the user dataset. By employing the MR-NLP technique and custom functions, we were able to extract meaningful insights and track usage trends. The implementation of predefined keywords and functions, such as “find_same_words” and “find_different_words,” facilitated accurate classification of commands, focusing on specific types. Additionally, the XML export feature further enhanced data compatibility and integration.

### Data analysis strategy

The data analysis strategy employed in this study aimed to assess the validity of three hypotheses concerning the efficiency, error rate, and accuracy of analytical interpretations derived from coding human-SVA speech interaction data by manual and automated coding methods.

To evaluate the hypothesis that rule-based NLP coding demonstrates higher efficiency compared to manual coding (H1), we measured the time required to code speech interaction data for one participant manually and then compared it with the time taken to process data for 35 participants using the rule-based NLP coding method. The efficiency of the NLP method was quantitatively assessed by calculating the percentage of time saved compared to manual coding.

To test the hypothesis regarding the error rate (H2), we conducted an inter-coder reliability analysis to compare the consistency between human coders and the rule-based NLP programmer. We calculated the agreement level and Cohen's kappa between the manual coding and the MR-NLP coding method, further breaking down discrepancies into categories of program errors, human errors, and differences in coding definitions.

The hypothesis concerning the accuracy of analytical interpretations (H3) was assessed by conducting exploratory factor analysis on a 20% subset of the data, coded using both manual and MR-NLP methods. This analysis aimed to identify whether the coding results led to divergent interpretations of user-SVA voice interactions. We performed principal-components factor analysis with varimax rotation to reveal the dimensions of user interactions across three datasets: one coded using the MR-NLP method post-discussion and discrepancy resolution, one using initial NLP coding, and one using manual coding. By comparing the factor structures and the variance accounted for by each method, we determined the impact of coding method choice on analytical interpretation.

## Results

### Hypothesis testing

*H1: rule-based NLP coding of human-SVA speech interaction data will demonstrate higher efficiency compared to manual coding of the same data*.

Manual coding took an average of 3 h to code one participant, with a range of 2–5 h, while the rule-based NLP coding method required 9 h to process 35 participants. The rule-based NLP coding method only consumed a small fraction of the time (9%) needed for manual coding. Consequently, H1 was supported.

*H2: modified rule-based NLP (Mr-NLP) coding of human-SVA speech interaction data will exhibit fewer unintentional errors than manual coding of the same data*.

To ensure consistency and agreement among the human coder and the rule-based NLP programmer in categorizing the commands, inter-coder reliability analysis was conducted. We employed the percentage agreement measure and Cohen's kappa to assess the agreement between coders. The percentage agreement calculates the percentage of agreement between coders by dividing the number of agreements by the total number of coding instances and multiplying by 100. Cohen's kappa adjusts for chance agreement between coders.

The agreement level between manual coding and the MR-NLP coding method was found to be 85.49% (872 cases) and Cohen's kappa was 0.82 based on a subset of 20% of the data. Among the 14.51% discrepancies (148 cases), 0.29% were attributed to program errors (3 cases) and 7.68% to human errors (67 cases). The remaining 6.54% (78 cases) resulted from differences in coding definitions, such as coding repeated commands for clarification by either Amazon Alexa™ or those participants with declining cognitive functions (25 or lower MoCA score). Consequently, H2 was supported.

Incorporating the feedback received, we extended our analysis by manually coding an additional 20% of the data, subsequently comparing these results with those generated by our NLP coding system. This comparative analysis revealed a remarkable consistency between human coding and NLP coding, with a 99% agreement rate and 0.99 Cohen's kappa in the classifications and interpretations of the data. Human error is the cause of the 1% disagreement due to human fatigue. This high degree of consistency underscores the reliability and accuracy of the NLP system in replicating human-like coding precision on a larger scale.

However, the efficiency comparison between the two approaches highlighted a significant advantage of the NLP system over manual coding. The human coder required approximately 20 h to complete the coding of the additional data subset. In stark contrast, the NLP system demonstrated its computational efficiency by processing the same amount of data in mere seconds. This substantial difference in processing time illustrates the profound impact of employing advanced NLP techniques in data analysis, offering a scalable and time-efficient alternative to traditional manual coding methods.

The findings from this expanded comparison not only address the initial concern regarding the model's comprehensiveness but also emphasize the NLP system's potential to provide accurate and efficient analysis of speech interaction data. Such results highlight the dual benefits of NLP coding: maintaining high accuracy in data interpretation while significantly reducing the time and resources required for data processing.

*H3: modified rule-based NLP (Mr-NLP) coding of human-SVA speech interaction data will result in more accurate analytical interpretations than manual coding of the same data*.

A crucial question for evaluating effectiveness of the two methods still remains: do the coding results from manual coding and the MR-NLP coding method lead to divergent analytical interpretations of the data, such as differences in data patterns and structures? It could be argued that if unintentional errors do not significantly impact the analytical interpretations, the case for strongly favoring one method over the other might not be as compelling as initially anticipated. To address this concern, an exploratory factor analysis was conducted three times on the 20% subset of data to summarize patterns of the 12 categories of daily routines and other interactions.

The test was performed under three conditions: first, using the final coding results from the MR-NLP coding method (data set 1); second, using the results from the NLP programmer before discussing and resolving discrepancies with the human coder (data set 2); and third, using the results from the human coder (data set 3). As all known errors and discrepancies were resolved in the final coding results using the MR-NLP coding method, the exploratory factor analysis results from data set 1 were considered the standard for comparison against the other two data sets (data set 2 and data set 3).

A principal-components factor analysis with varimax rotation was employed to test the factor structure of the 12 items. We employed factor analysis as a method to distill the complex interactions within the dataset into more manageable, interpretable dimensions. This statistical technique helps in identifying underlying variables, or factors, that explain the pattern of correlations among the observed variables. In the context of our study, it allowed us to systematically reduce the dimensionality of the coding results, thus making the data's structure more comprehensible. By doing so, we aimed to uncover the fundamental constructs that characterize user-SVA interactions, providing a robust means to compare the analytical interpretations derived from both manual and NLP coding methods.

The analysis revealed the presence of four dimensions of user-SVA voice interactions, accounting for 92% of the total variance of the items in data set 1 using the MR-NLP coding method (see Table 1). Similarly, the principal-components factor analysis test performed using data set 2 from the initial rule-based NLP coding method indicated four dimensions as well, accounting for 91.69% of the total variance (see Table 2). However, results of the principal-components factor analysis test from data set 3, which was manually coded by a human, suggested five dimensions among the 12 items (see Table 3). Therefore, unlike the variations of NLP coding methods used in data sets 1 and 2, human coding led to a different interpretation of the data pattern in data set 3. As a result, H3 was supported.

**Table 1:** Factor loadings using principal component and varimax rotation in data set 1 using the modified rule-based NLP model.

	1	2	3	4
Good morning	.377	.874	−.079	.057
Big sky	−.174	−.175	.889	.168
Riddle	.033	.908	−.089	.272
Five minute morning meditation	.335	.212	.832	.235
Music	.675	.453	.514	.230
Good afternoon & evening	.655	.399	.210	.601
Weather	.768	.493	−.363	−.073
Joke	.889	.251	−.126	−.040
Akinator	.528	.263	.048	.780
Calls	.125	.739	.423	−.336
Goodnight	.178	.113	−.386	−.832
Setting volume & speed	.876	−.114	.297	.146
Eigenvalue	5.57	2.71	1.51	1.22
Proportion of variance	46.40%	22.59%	12.56%	10.15%

Total eigenvalue = 11.01. Total proportion of explained variance = 91.70%.

**Table 2:** Factor loadings using principal component and varimax rotation in data Set 2 by the programmer using the initial rule-based NLP model.

	1	2	3	4
Good morning	.373	.877	−.067	.061
Big sky	−.195	−.183	.878	.172
Riddle	.910	−.084	.261	.910
Five minute morning meditation	.316	.205	.839	.243
Music	.634	.480	.550	.210
Good afternoon & evening	.641	.399	.222	.610
Weather	.778	.497	−.339	−.072
Joke	.891	.253	−.110	−.028
Akinator	.518	.264	.059	.786
Calls	.119	.736	.437	−.335
Goodnight	.196	.115	−.381	−.829
Setting volume & speed	.867	−.113	.315	.157
Eigenvalue	5.56	2.72	1.52	1.21
Proportion of variance	46.31%	22.63%	12.64%	10.12%

Total eigenvalue = 11.01. Total proportion of explained variance = 91.69%.

**Table 3:** Factor loadings using principal component and varimax rotation in data set 3 coded manually by human coder.

	1	2	3	4	5
Good morning	.961	.145	.202	.041	−.009
Big sky	−.062	−.318	−.855	.222	.070
Riddle	.791	.309	−.059	−.395	.262
Five minute morning meditation	−.711	−.047	.626	−.127	−.162
Music	.578	.205	−.265	.703	.240
Good afternoon & evening	−.024	−.966	−.134	−.030	−.087
Weather	−.061	.047	.917	.372	−.029
Joke	.199	.898	.139	.304	−.033
Akinator	.132	.381	−.031	.241	.882
Calls	.918	−.099	−.121	.294	−.150
Goodnight	.077	.538	.151	−.119	−.816
Setting volume & speed	.011	.182	.150	.947	.214
Eigenvalue	4.15	2.95	2.06	1.37	1.21
Proportion of variance	34.55%	24.57%	17.18%	11.39%	9.34%

Total eigenvalue = 11.74. Total proportion of explained variance = 97.03%.

## Discussion

### Manual coding vs. NLP coding

NLP has emerged as a powerful tool for analyzing and processing human language data. As demonstrated by the results in this study, when applied to examining older people's speech interactions with SVA, rule-based NLP coding offers numerous benefits over manual coding, including increased efficiency and reduced unintentional errors. The MR-NLP algorithms utilized in this study processed text data at a significantly faster pace compared to manual annotation. It is important to highlight that rule-based NLP algorithms can seamlessly process theoretically unlimited amounts of additional data within seconds once the rules have been finalized. On the other hand, human coders will always require additional time to code data from new participants.

As the dataset grows, manual coding becomes increasingly challenging to manage and may not be scalable for handling extensive or complex data. In contrast, similar to NLP's scalability in business applications (35), the time- and cost-saving advantages of the proposed MR-NLP coding for older people's speech interactions with SVA are exponential. This efficiency gain will free up valuable time and resources for researchers, enabling them to focus more on discovering insights and providing valuable assistance to older individuals in their journey of aging well.

Our data further supported the notion that NLP coding can effectively reduce unintentional errors compared to manual coding. The human coder in our study made these unintentional errors primarily due to fatigue and the repetitive nature of processing unstructured text data downloaded from SVA. Coder fatigue is a recognized threat to the trustworthiness of content analysis, particularly in cases involving repetitive and clerical coding (36). The results obtained while testing H3 demonstrated that even unintentional human errors can lead to misinterpretations of the data patterns.

Through our study, we have provided a valuable solution to prevent such mistakes in future studies involving text-based human-SVA interaction data. By implementing rule-based NLP coding, researchers can mitigate the impact of unintentional errors caused by fatigue and repetitive coding, thus enhancing the accuracy and reliability of content analysis.

Nonetheless, human feedback has proven invaluable in providing recommendations to improve the rules for NLP algorithms in the present study. When the human coder and the rule-based NLP programmer discussed their discrepancies on the data categorized by routines and other interactions, coding repeated commands for clarification emerged as a primary source of divergence. This observation led to the revelation of the context surrounding such speech repetitions, specifically the relationship between declining cognitive functions and repeated commands by participants. Without human input, this crucial contextual understanding of older people's speech interactions with SVA might have been overlooked. It's evident that integrating manual and automated coding methods into a MR-NLP model is essential for analyzing the voice interaction data between older adults and smart voice assistants.

Consistent with previous research (20, 21), the speech interactions between older people and SVA appear to be influenced by cognitive decline, resulting in repetitions in their speech patterns. These characteristics present challenges when processing qualitative human-SVA speech data. From a user experience (UX) design standpoint, it is crucial for researchers and SVA providers to adapt their AI algorithms to accommodate older people's changing speech patterns caused by cognitive impairment. By doing so, they can enhance the overall flow of conversation between the user and the SVA. The valuable insights obtained from our study can significantly contribute to the development of more inclusive and accessible technologies tailored to the needs of older individuals. Implementing these design improvements will ensure that older people can interact with SVAs more effectively and comfortably, ultimately leading to a more positive and enriching user experience for this demographic.

### Four-factor solution vs. five-factor solution

The divergent factor analysis outcomes observed between the human-coded data and the NLP-coded data warrant a detailed examination. The principal difference between the four- and five-factor solutions lies in how they conceptualize and categorize the interactions. The four-factor solution, derived from the MR-NLP coding method, suggests a more consolidated view of interaction categories, likely reflecting the algorithm's capacity to group similar interactions based on predefined rules and learning from the coder-NLP programmer discussions. On the other hand, the five-factor solution, emerging from the manual coding, indicates a finer granularity in categorizing interactions. This additional factor could suggest either a unique dimension of interactions captured by human coders but not by the MR-NLP model or potential noise introduced by human error or subjectivity in interpreting interactions.

Regarding the fifth factor, particularly its association with Akinator interactions, it's crucial to clarify that this doesn't solely represent noise or an error. Akinator interactions could indeed reflect a distinct category of engagement with SVAs not fully captured or appropriately grouped by the NLP model. This discrepancy underscores the nuanced understanding human coders have about context and the dynamic nature of human-SVA interactions. It also highlights the challenge in designing NLP models that fully encapsulate the breadth of human interaction nuances. However, without further analysis, we cannot definitively label this fifth factor as noise; it warrants additional investigation in future studies.

One main source of human errors came from the “Akinator” item, particularly due to overcounting interactions due to misinterpretation of repeated keywords or phrases as separate interactions. However, it wasn't the sole source of human errors. These were broadly related to the challenges in consistently applying the coding rules to varied interactions, indicating areas for further training and refinement in manual coding practices.

### Cognitive functioning of older people

Our use of the MoCA was intended purely for descriptive purposes, to outline the cognitive functioning profiles within our participant group, rather than to diagnose or classify participants according to their cognitive status. Given the limitations of our sample size, direct comparisons or analyses stratified by specific MoCA score ranges were not feasible. However, the diversity in cognitive functioning among our participants, as indicated by their MoCA scores, suggests potentially meaningful inferences about the broader implications of cognitive decline on technology interaction patterns. Specifically, our findings highlight how interaction behaviors—such as command repetitions, corrections, and modifications—may mirror adaptive strategies employed by older adults to navigate potential cognitive challenges. These observed behaviors provide a window into understanding how SVAs might be used as supportive tools for this demographic, potentially offering indirect insights into users’ cognitive states.

Furthermore, the study opens avenues for future research to explore the relationship between cognitive functioning levels, as broadly indicated by MoCA scores, and SVA interaction patterns. While our sample size and study design do not support direct comparisons across different cognitive levels, our methodology and findings lay the groundwork for subsequent investigations. Such research could aim to identify specific patterns of SVA use that correlate with varying degrees of cognitive functioning, thereby enhancing our understanding of how technology can be optimized to support older adults across the cognitive spectrum.

Regarding the implications of our findings within the context of cognitive decline, it is pertinent to acknowledge that our primary objective was to showcase the utility of the MR-NLP model for analyzing older adults’ voice interactions with SVAs. Nevertheless, the exploratory insights derived from our study—facilitated by the inclusion of MoCA scores for descriptive purposes—suggest potential areas for further investigation. Future studies, with larger and more diverse samples, are encouraged to explicitly examine the impact of cognitive functioning on technology use among older adults. Such research could significantly contribute to the development of SVAs and other technologies that are more responsive and tailored to the needs of older individuals with varying cognitive abilities.

## Limitations

While our investigation of rule-based NLP coding for older people's speech interactions with SVA provides compelling evidence of its efficiency gains and error reduction, it is important to acknowledge its limitations.

### Implications of partial data coding by human coder

In this study, we opted to manually code a 20% subset of the data derived from interactions between older adults and SVAs, acknowledging the potential limitations this approach might entail. While this strategy was adopted to balance depth with feasibility—given the labor-intensive nature of manual coding—it is essential to consider what might have been overlooked by not coding the entirety of the data.

Firstly, coding only a portion of the data could result in missing out on less frequent, yet potentially significant, patterns of interaction that could offer deeper insights into user behavior or uncover unique use cases of the technology. These rare instances might reveal innovative ways in which SVAs are utilized by older adults, offering valuable perspectives on user adaptability and the technology's versatility. Secondly, the variability in daily routines and the specific commands used by participants might not be fully captured. This variability can provide insights into personal preferences, adaptability to technology, and even changes in cognitive abilities over time. By not examining the full dataset, nuances related to how different individuals interact with SVAs across various contexts might have been underrepresented.

To mitigate these limitations, we employed rule-based NLP coding, designed to extrapolate the insights gained from the manually coded subset to the larger dataset. This approach, while efficient, relies on the assumption that the sampled data adequately represent the broader dataset. In future iterations of this research, investigators should aim to explore methods that combine the efficiency of NLP with the depth of manual coding, potentially through adaptive sampling techniques that allow for more dynamic selection of data for manual review based on preliminary findings.

### Sample and system applicability

The study's findings are grounded in data collected from a specific sample of older adults, characterized by their age, living situation, and engagement with SVAs. While our sample provides valuable insights into how this demographic interacts with technology, it is crucial to discuss its broader applicability. Our participants were predominantly white, English-speaking older adults residing in independent living facilities within a specific region. This demographic profile raises questions about the generalizability of our findings to other groups, including individuals from diverse ethnic backgrounds, non-English speakers, or those with different living arrangements such as those residing with family or in assisted living facilities.

Furthermore, the cognitive functioning of our participants, as assessed by the MoCA, varied, indicating a range of capabilities in interacting with technology. This variability is reflective of the broader older adult population, yet our findings may not fully encapsulate the experiences of individuals with more severe cognitive impairments or those who are technologically adept.

We acknowledge these limitations and propose avenues for future research. Specifically, studies involving more diverse samples, including varying ethnic backgrounds, languages, and cognitive abilities, are essential to understand the full spectrum of older adults’ interactions with SVAs. Additionally, exploring the impact of different living arrangements on SVA usage could offer further insights into how these technologies can support independent living and social connectivity among older adults. By broadening the scope of research in this area, we can better tailor SVA technologies to meet the diverse needs of the older adult population, thereby enhancing their usability and effectiveness in supporting aging well.

Like any other NLP algorithms, the performance of our model heavily relies on the quality and representativeness of the training data. For future studies, it is advisable to consider a larger and more diverse sample of participants. This will enable the NLP algorithms to achieve higher accuracy when predicting patterns within the growing and increasingly diverse aging population.

Furthermore, it is essential to note that our current algorithms are programmed to process English-language text data. However, it is entirely feasible to modify and adapt them to handle text data in different languages. By doing so, researchers can extend the benefits of NLP coding to a broader global audience and facilitate cross-lingual analysis of human-SVA interactions among older individuals.

Our data were collected after the lifting of COVID-19 restrictions in most cities in the United States. However, it is possible that the impact of the COVID-19 pandemic persisted during our study. Older people have reported an increased usage of and a more positive attitude toward digital technology, including SVA, during the COVID-19 pandemic (37). Nevertheless, it remains uncertain if such trends will continue in the future. We encourage replications of our study to explore the application of rule-based NLP coding for older people's SVA interactions under different circumstances.

### Future comparative analysis

While our study has demonstrated the effectiveness of the MR-NLP model in comparison to manual coding, there are certain limitations that should be acknowledged. First, the primary comparison in our study was between MR-NLP and manual coding processes. While this comparison effectively highlighted the improvements in efficiency and reduction of errors afforded by MR-NLP, it did not encompass comparisons with other automated NLP models. Consequently, the results are focused on demonstrating the superiority of MR-NLP over traditional manual methods, rather than a broad spectrum analysis against contemporary automated systems. Second, the scope of this study was intentionally focused on validating the MR-NLP model against manual coding to establish a baseline of its performance. While this focus was crucial for the initial validation of the model, it limits the understanding of how MR-NLP performs in the broader landscape of NLP technologies where various automated models are employed. Third, although our study lays a solid groundwork for the use of MR-NLP in practical settings, including an open access web app and detailed protocol for replication, it suggests a need for future studies to undertake a comparative analysis with other rule-based and machine learning-based NLP models. Such comparisons would be invaluable in rigorously assessing the scalability and overall efficiency of MR-NLP against other models in handling diverse and large datasets. Fourth, while we have provided all necessary tools and protocols for other researchers to replicate our work, and demonstrated MR-NLP's operational efficiency, the full scalability of our model remains to be tested across varied contexts and larger datasets. Future research could use our published resources to validate and extend our findings in different settings, which would provide deeper insights into the applicability and scalability of MR-NLP. By acknowledging these limitations, we invite the scientific community to both utilize the MR-NLP framework in their work and to build upon our initial findings through further comparative and scalability studies. This ongoing research will be crucial in refining the application of NLP technologies in real-world settings and ensuring that these tools meet the evolving needs of diverse populations.

In summary, the proposed MR-NLP model proves to be an effective tool for investigating older people's speech interactions with SVA or other AI technology. This innovative software solution can be replicated by other researchers by following the outlined procedures using open source software. An online version of our proposed method can be accessed with permission via https://excel-helper-deploy.vercel.app. By providing this method to the research community, we hope to facilitate further exploration and advancement in the development of NLP models, ultimately contributing to a better understanding of the dynamics between older people and AI-based technologies.

## Data Availability

The raw data supporting the conclusions of this article will be made available by the authors, without undue reservation.
