# Supplementary material for: Understanding older people's voice interactions with smart voice assistants: a new modified rule-based natural language processing model with human input
Source: Front Digit Health. 2024 May 14;6:1329910. doi: 10.3389/fdgth.2024.1329910 (PMC11135128; doi:10.3389/fdgth.2024.1329910)
Supplement: Supplementary file 1 [file Datasheet1.pdf]

Appendix 1: Detailed Descriptions of Pre-selected Daily Routines for Older People's Voice Interactions with Personal Voice Assistants (PVAs)

1. Greetings: Participants were instructed to initiate their day with Alexa by saying "Good Morning," and similarly, to use "Good Afternoon," "Good Evening," and "Goodnight" at the appropriate times of day, fostering a natural interaction with the device as one would do with a family member or a friend.

2. Big Sky: This involved asking Alexa for detailed weather forecasts, tapping into Alexa's ability to provide hyper-local weather information, which is a frequent use case for PVA users.

3. Daily Riddle: Engaging with Alexa to solve a riddle, adding an element of fun and mental stimulation to the daily routine.

4. Five Minute Morning Meditation: Participants were directed to start their mornings with a brief meditation session via Alexa, demonstrating the device's capability to support wellness and mindfulness practices.

5. Music: Instructions included asking Alexa to play music, whether it be specific songs, artists, or genres, showcasing the assistant's integration with various music streaming platforms and its role in personal entertainment.

6. Weather: Beyond the Big Sky interaction, participants were also asked to request general weather forecasts, a testament to the routine reliance on PVAs for daily planning.

7. Asking for a Joke: This activity aimed to explore Alexa's ability to generate entertainment and engagement through humor, providing a light-hearted interaction.

8. Playing the Akinator Guessing Game: Participants engaged with Alexa in a game of Akinator, where the device guesses a character the user is thinking of through a series of questions, highlighting the AI's interactive and entertainment capabilities.

9. Calls to Existing Social Connections: Participants made use of Alexa's communication functions to make calls, illustrating the device's role in maintaining and facilitating social connections.
