# Supplementary material for: Understanding older people's voice interactions with smart voice assistants: a new modified rule-based natural language processing model with human input
Source: Front Digit Health. 2024 May 14;6:1329910. doi: 10.3389/fdgth.2024.1329910 (PMC11135128; doi:10.3389/fdgth.2024.1329910)
Supplement: Supplementary file 2 [file Datasheet2.pdf]

Appendix 2: A Comprehensive Coding Schema for Coding Older People's Voice Interactions with Personal Voice Assistants (PVAs)

1. Overview
- Purpose: To categorize and analyze voice interactions of older people with PVAs.
- Interaction Categories: 12 predefined categories.

2. Predefined Categories
Each interaction is classified into one of the following categories:
1. Morning Greetings: Interactions involving general greetings like "Good Morning," "Morning, Alexa."
2. Afternoon and Evening Greetings: Interactions involving general greetings like "Good Afternoon," "Good Evening," and "Goodnight."
3. Big Sky: Inquiries or commands related to Big Sky, pertaining to detailed weather information.
4. Daily Riddle: Engagements that involve asking for or solving a daily riddle.
5. Five Minute Morning Meditation: Requests or initiations of a brief morning meditation routine.
6. Music: Interactions involving playing music, song requests, or inquiries about musical content.
7. Weather: General weather-related questions or commands, distinct from Big Sky for its broader scope.
8. Asking for a Joke: Requests to hear a joke, typically for entertainment purposes.
9. Playing the Akinator Guessing Game: Interactions related to playing the Akinator game.
10. Calls to Existing Social Connections: Voice commands or queries related to making phone calls to known contacts.
11. Setting Adjustments: Adjustments to the voice assistant's settings, including volume, language, accessibility features, etc.
12. Other Routines: Voice interactions that involve establishing, querying, or executing routines not covered in the above categories.

3. Coding Rules
- Specific Keywords/Phrases within an Uninterrupted Interaction: Identify and use a specific keyword or phrase indicative of each category in an uninterrupted interaction. Repeated keywords or phrases in a single uninterrupted interaction should be counted as one interaction.
- Context Consideration: Understand the context of the interaction to accurately categorize it.
- Multiple Categorizations: If an interaction spans multiple categories, code it under all applicable categories. For example, "Good Morning, Play Music" will be coded as both morning greetings and music categories.
- Review for Ambiguities: Review interactions categorized as "Miscellaneous" for potential inclusion in existing categories.

4. Data Management
- Recording Method: Utilize a structured format like an EXCEL spreadsheet for data entry.
